# A Leakage Prediction Model for Sealing Performance Assessment of EPDM O-Rings under Irradiation Conditions

**DOI:** 10.3390/polym15143073

**Published:** 2023-07-17

**Authors:** Xiaoming Huang, Jimin Gu, Ming Li, Xinli Yu, Yu Liu, Guoliang Xu

**Affiliations:** 1 School of Energy and Power Engineering, Huazhong University of Science and Technology, Wuhan 430074, China; xmhuang@hust.edu.cn (X.H.); m202171261@hust.edu.cn (J.G.); lm001203@163.com (M.L.); 2 China Nuclear Power Engineering Co., Ltd., No.117, Xisanhuanbeilu, Haidian District, Beijing 100840, China; yuxl@cnpe.cc (X.Y.); 13946029652@163.com (Y.L.)

**Keywords:** O-ring, irradiation, leakage rate, prediction model, sealability assessment

## Abstract

In this work, a model for predicting the leakage rate was developed to investigate the effect of irradiation on the sealing performance of ethylene propylene diene monomer (EPDM) O-rings. The model is based on a mesoscopic interfacial gap flow simulation and accurately predicts the sealing performance of irradiated and non-irradiated materials by utilizing the gap height as an indicator in a mechanical simulation of the O-ring under operating conditions. A comparison with vacuum test results indicates that the model is a good predictor of leak initiation. The positive pressure leakage of the O-rings was investigated numerically. The results show the following. The sealing performance of the non-irradiated O-ring is much better than that of the irradiated one. The sealing performance is the worst at 0. 713 MGy and the best at 1.43 MGy, and the seal is maintained at an absorbed dose of 3.55 MGy. A theoretical analysis of the non-monotonic variation using the proposed model shows that the leakage behavior of the O-rings depends not only on the material properties but also on the roughness and prestressing properties. Finally, a method was proposed to classify the sealing performance, using the maximum allowable leakage rate as an indicator.

## 1. Introduction

Due to their excellent elasticity and resilience, rubber seals are widely used in nuclear power plants (NPPs) as an important component of the containment pressure boundary [1]. However, as with all polymers, the sealing properties of rubber seals can degrade when they are exposed to oxidation, heat, and irradiation [2,3,4]. If significant leakage occurs, the integrity of the NPP containment could be breached, resulting in catastrophic consequences for the NPP and the surrounding area [5].

Rubber seals must withstand high radiation doses during an accident. Irradiation studies of rubber materials are widely used for assessing their reliability in accidents [6,7,8]. Ethylene propylene diene monomer (EPDM) has attracted the most attention due to its excellent irradiation resistance [9,10,11,12,13]. Le Lay et al. [9] used experimental measurements to investigate the effects of gamma irradiation at doses of 10, 20, 250, and 400 kGy on the mechanical properties of EPDM. At low doses (corresponding to normal nuclear plant operating conditions), EPDM can be used for more than 32 years, with negligible changes in its properties. Plaček et al. [10,11] investigated the lifetime of two EPDM seals with 7 mm and 14 mm thicknesses during the simulation of severe accidents in an NPP. They did not use the compression set (CS) as a lifetime criterion but performed a gas leakage test at the operating pressure to obtain the seals’ lifetimes. Nakano et al. [12,13] conducted several studies on the irradiation resistance of different rubber materials at doses ranging from 100 kGy to over 3 MGy. They tested the hardness, tensile strength, elongation at break, compression set, and sealability at the operating pressure. EPDM had the highest irradiation resistance, which depended on the material’s inorganic content. Battini et al. [14] and Ferrari et al. [15] conducted several studies on the degradation of EPDM elastomers irradiated at high dose rates in mixed neutron and gamma fields and found that material degradation was mainly influenced by the total absorbed dose as opposed to the irradiation type and irradiation dosage.

The elongation at break and compression set (CS) are preferred indicators of the degree of irradiation degradation of sealing materials [10,12,14]. The elongation at break reflects the tensile properties of the material; a lower value indicates that the material is more brittle and prone to fracture. The compression set reflects the resilience of the material after the load has been released; the higher the value, the worse the sealing properties of the material. However, a growing number of researchers have found no direct correlation between these mechanical property parameters and the sealing performance of the sealing material [10,14,16]. For example, the leakage rate of a gasket with a complete loss of elasticity (CS above 100%) is still within the acceptable range [16]. The results of material irradiation damage studies are of limited use in creating safe designs or the risk assessment of sealing products in an accident since information on the leakage amount is not available.

Finite element analysis has been increasingly used to study the effects of irradiation or thermal ageing of seals and the sealing performance. Several researchers have numerically investigated the hyperelastic properties of irradiated rubber materials and evaluated the validity of different constitutive models [17,18,19]. In addition, mechanical and viscoelastic property parameters obtained from experimental measurements [20] have been used to numerically simulate compression set tests of rubber materials at different temperatures. Finite element techniques can minimize or eliminate experimental testing and extend the use of experimental data. Similarly, finite element simulations do not provide information on leakage rates, and, therefore, their results cannot be directly related to the sealing performance of the sealing material.

In addition to the material’s mechanical properties, the sealing performance of an installed seal is influenced by many factors, including the geometry of the seal, the applied sealing force, and the pressure of the sealing medium. The leakage rate is the most widely used and direct indicator of the seal’s reliability used in a containment. Some researchers have attempted to correlate the leakage with material degradation, using experiments [21,22,23]. However, these empirical correlations are often not scalable and are only applicable to a very narrow context. Other researchers have utilized theoretical leakage rate models for sealing interfaces [24,25]. The main cause of fluid leakage is the presence of fine leakage channels at the sealing interface. Existing leakage models can be broadly classified into three categories based on the characterization of the leakage channels: ideal channel [26,27], multiscale fractal [28,29], and morphological models [30,31,32]. The ideal channel model, represented by the Roth model, regards the leakage channel as a microchannel with a regular geometry (e.g., triangular or circular) and equal cross-section. This model is simple but has low accuracy. The fractal model considers the multiscale characteristics of the leakage channel, but some characteristic parameters related to the fractal scale cannot be easily obtained, especially when time, temperature, and environmental effects must be considered. In contrast, morphological models are based on numerical reconstruction techniques of rough surfaces and accurately reflect the complexity and tortuosity of leakage channels without requiring physically ambiguous physical parameters. Although these models have not been unified in terms of channel morphology characterization or gap flow description, they provide new ideas for studying the performance of seals. Predictive models reveal the relationship between various factors and the leakage rate and enable the assessment of the seal’s reliability, using the leakage rate as a direct indicator. The interfacial leakage rate has not been used to date to predict and evaluate the leakage behavior of seals due to ageing and degradation.

Our previous work proposed a morphology-based approach to predict the leakage rate by coupling several numerical techniques [31,32]. A numerical reconstruction of rough surfaces was used to simulate machined surface roughness and assess leakage channels at the sealing interface. To better reflect the effect of surface roughness on fluid flow, the D3Q19 lattice Boltzmann method (LBM) was chosen to determine the gas flow at different pressure differentials at mesoscopic scales. A theoretical model of the interfacial leakage was constructed based on the numerical results, incorporating the effects of geometry, surface roughness, and interfacial gap height. Since this highly scalable model was not based on empirical relationships, it was used to study the sealing performance of fuel cell seals and predict the long-term leakage characteristics of the seals with different environments, compression rates, and roughness surfaces.

In this work, a similar approach was used to model the interfacial leakage of EPDM O-rings. A numerical approach based on the model was developed to evaluate the irradiation effect on the O-rings’ sealing performance, using the leakage rate as an indicator. The mechanical deformation behavior of the O-rings exposed to different irradiation levels is obtained by finite element simulations in conjunction with measurements of the material’s mechanical properties. Macroscopic simulations of the O-ring deformation and mesoscopic simulations of the interfacial leakage are conjuncted by using the interfacial gap height as a critical parameter. Simulations of the micro-indentation process with multiple asperities are performed to establish the quantitative relationship between gap height and contact stress.

Based on the proposed approach, the contact state, leakage behavior, and safety assessment of the O-ring exposed to different irradiation doses are discussed in detail to guide the design of safe EPDM O-rings used in NPP containment.

## 2. Leakage Prediction Model of O-Ring

### 2.1. Mesoscopic Simulation of Interfacial Leakage

**Figure 1:**
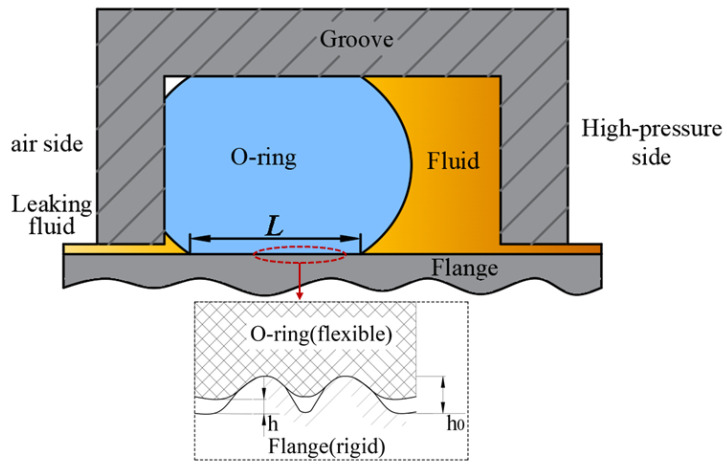
Illustration of the leakage mechanism of an O-ring.

O-ring seal assemblies are widely used in containment penetrations. The leakage mechanism is illustrated in Figure 1. Many small leak paths occur between two contacting rough surfaces. After the application of the sealing force, *F*, the compressed elastomer seal fills part of the leakage path through elastic deformation, preventing fluid leakage.

**Figure 2:**
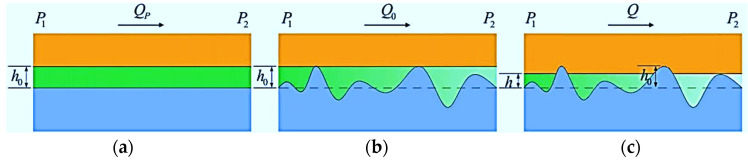
Definition of the three leakage channels and their leakage flows: (**a**) smooth interface gap, (**b**) rough interface gap, and (**c**) sealing interface gap.

The three gap models (Figure 2) can be used to investigate the effects of the roughness and sealing force on the interface leakage. If the contact interface has a gap between the two smooth surfaces with a separation distance, *h*_0_ (Figure 2a), the flow rate can be calculated using the Poiseuille equation:

(1) $$Q_{P}=\frac{Bh_{0}^{3}}{12\;\mu L}\frac{P_{1}^{2}-P_{2}^{2}}{2P_{1}}$$

where $Q_{P}$ is the volumetric flow rate; *L* is the interface length, *B* is the interface width; *h*_0_ is the nominal height of the interface; *μ* is the dynamic viscosity of the fluid; and $P_{1}$ and $P_{2}$ are the inlet and outlet pressures, respectively.

The sealing surface in Figure 2b has a machined roughness. The roughness flow factor, $\Phi_{\sigma }$, is defined as follows to characterise the effect of the roughness $\left(\sigma ,T\right)$ on the gap flow:

(2) $$\Phi_{\sigma }=\frac{Q_{0}}{Q_{P}}=f_{\sigma }\left(\sigma^{*}\right)$$

where $Q_{0}$ is the gap flow rate for a rough interface with the same gap height, $h_{0}$, as $Q_{P}$. Since the roughness affects the difference between $Q_{0}$ and $Q_{P}$, $\Phi_{\sigma }$ depends only on the dimensionless roughness parameter, $\sigma^{*}$. The dimensionless roughness, $\sigma^{*}$, is defined as the ratio of the root mean square roughness, *σ*, of the Gaussian rough surface to the autocorrelation length, *T*: $\sigma^{*}=\sigma /T$.

When a sealing force is applied, the rough interface is deformed by squeezing, reducing the gap height, as shown in Figure 2c. A height flow factor, $\Phi_{h}$, is defined to characterize the effect of the gap height on the gap flow as follows:

(3) $$\Phi_{h}=\frac{Q}{Q_{0}}=f_{h}\left(h^{*}\right)$$

where Q is the gap flow after the application of the sealing force, reflecting the leakage rate through the sealing interface. Q and $Q_{0}$ have identical roughness but different gap heights. Therefore, $\Phi_{h}$ depends only on the dimensionless height, $h^{*}$. The dimensionless gap height, $h^{*}$, is the ratio of the gap height, *h*, at the sealing interface to the gap height, $h_{0}$, at the rough interface: $h^{*}=h/h_{0}$.

The model for calculating the leakage rate at the O-ring interface using the two flow factors can be expressed as follows:

(4) $$Q=\Phi_{\sigma }\Phi_{h}Q_{P}$$

Simulations of the interface flow are carried out at the mesoscopic scale to derive the equations for the two flow factors in Equation (4).

**Figure 3:**
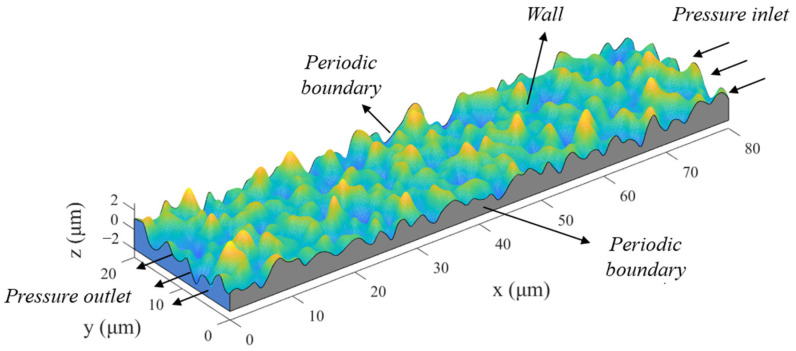
Simulation model of leakage flow channels for a rough interface gap.

**Figure 4:**
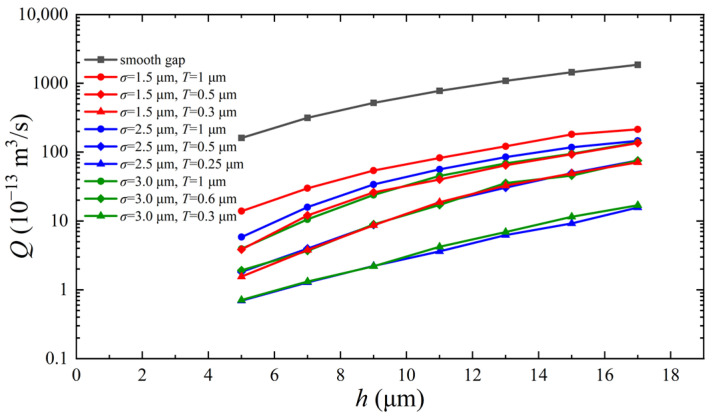
Calculated leakage rate for surfaces with different roughness values.

A numerical analysis of Gaussian rough surfaces is used to construct flow passages with different roughness values at the interface, as shown in Figure 3. The top surface is the generated rough surface, and the bottom surface is a smooth surface; both are solid wall surfaces with no flow. The x-direction is the flow direction of the leaking medium, and the two ends are the pressure inlet and pressure outlet, respectively. The y-direction is the periodic boundary at both ends. The interfacial leakage is assumed to have isothermal incompressible flow. The simulation of the leakage flow uses the D3Q19 LBM with the lattice Bhatnagar–Gross–Krook (LBGK) collision operator to reflect the influence of the rough wall surface on the microflow. The calculations were implemented in the commercial software MATLAB, and the detailed steps and methodology are similar to those in our previous work [32]. Since the method description is lengthy, it is not repeated here, and only the simulation results are shown (Figure 4).

The simulation results correspond to ten rough interfaces (including one ideal smooth interface). Different heights of the sealing interface are simulated by moving the position of the smooth surface. Seven gap heights were evaluated for each rough interface. The roughness flow factor, $\Phi_{\sigma }$, and the height flow factor, $\Phi_{h}$, were calculated as follows:

(5) $$\Phi_{\sigma }=0.9257{\left(\sigma^{*}\right)}^{-1.505}$$

(6) $$\Phi_{h}=0.03021e^{3.556h^{*}}$$

**Figure 5:**
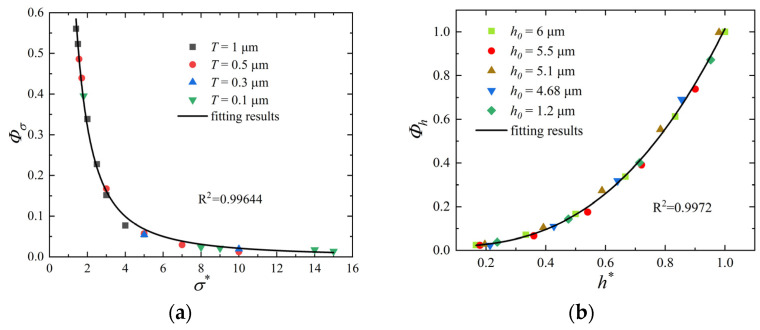
Simulation results and fitted curves for the two flow factors: (**a**) $\Phi_{\sigma }-\sigma^{*}$ curve and (**b**) $\Phi_{h}-h^{*}$ curve.

The deviations between the calculated values of the two factors and the fitted values are shown in Figure 5.

### 2.2. Assessing the Gap Height at the Sealing Interface

The effect of the sealing force on the height of the gap, *h*, at the interface depends on the micro-contact deformation of the rough surfaces. Greenwood and Williamson [33] first proposed a multi-asperity contact model for the contact of two rough surfaces which has been widely used. In their model, the roughness of a surface is characterized by asperities with a statistical distribution of the peak heights and a common radius of the curvature. The asperities are in elastic contact and do not interact. Therefore, the contact deformation, *δ*, for a single asperity under the mean contact stress, *S_G_*, can be solved by applying Hertz’s elastic contact theory [34]:

(7) δR=9SG216E′213

where *S_G_* is the apparent mean contact stress in MPa, and E′ is the composite modulus of elasticity of the two materials. Equation (7) indicates that the dimensionless deformation, *δ/R*, of the asperity is a function of the dimensionless contact stress, $S_{G}/E\prime$.

**Figure 6:**
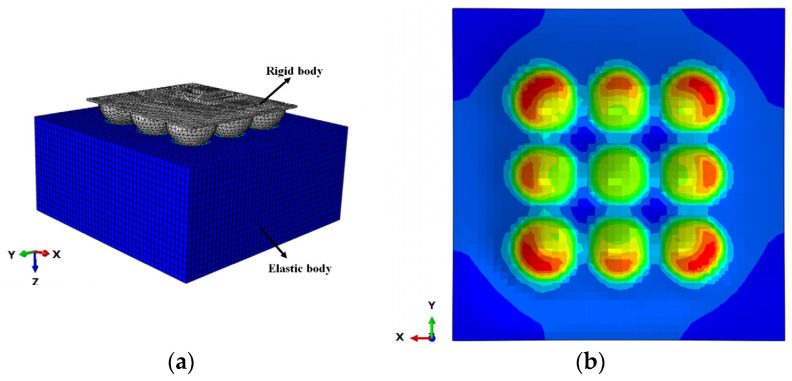
Finite element model of the micro-indentation process with multiple asperities: (**a**) schematic diagram of the grid and (**b**) stress distribution diagram.

The interaction between asperities is not considered in the Greenwood and Williamson model. However, the effect of this interaction on contact deformation may be significant for rubber seals [35]. Therefore, the micro-contact deformation of a 3 × 3 array of multiple asperities was numerically investigated using the commercial software package ABAQUS to derive the quantitative relationship between the contact stress and the gap height. The finite element analysis model is shown in Figure 6. The upper part is a rigid surface with multiple asperities, and the lower part is an elastic semi-infinite body with a smooth surface.

The asperities have the same radius of curvature, $R=h_{0}$. The indentation process is simulated using the explicit solver with a hexahedral mesh. The bottom of the elastomer is fixed, and the sides are symmetrically constrained. A displacement load in the *z*-axis direction is applied to the rigid body with multiple asperities, and the displacement is the indentation depth of the asperities, *δ*. The gap height, *h*, at the sealing interface is obtained by the following equation:

(8) $$h=h_{0}-\delta$$

**Figure 7:**
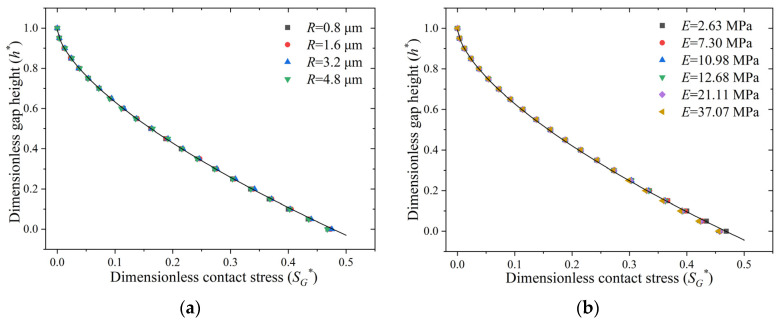
Simulation results of the relationship between $h^{*}$ and $S_{G}^{*}$: (**a**) effect of curvature radius and (**b**) effect of Young’s modulus.

Equation (7) indicates that the results of the finite element analysis can be simplified to a relationship between the dimensionless gap height, $h^{*}$ ($h^{*}=h/h_{0}$), and the dimensionless contact stress, $S_{G}^{*}$. Figure 7 shows the calculated results of $S_{G}^{*}-h^{*}$ for asperities (Figure 7b) with different curvatures (Figure 7a) and different elastic moduli and the fitted curves. The roughness and modulus have negligible effects on the relationship between $S_{G}^{*}$ and $h^{*}$ in the examined range. The relationship between $S_{G}^{*}$ and $h^{*}$ is obtained by fitting the calculated results in Figure 7 as follows.

(9) $$h^{*}={-1.604S_{G}^{*}}^{0.6487}+0.9928$$

Equations (4)–(6) and (9) describe the quantitative relationship between the interfacial leakage rate and the contact stress and contact width. Although this quantitative relationship is obtained on a mesoscopic scale, it is scale-independent due to the dimensionless form, and the physical mechanism is clear, enabling the extension to macroscopic scales. As long as the contact stress, *S_G_*, and contact width, *L*, at the seal interface are known for a given service condition, the model can be used to predict the interface leakage rate. In this work, the finite element analysis was used to determine the contact conditions of irradiated and non-irradiated O-rings in service and predict their interface leakage patterns.

## 3. Numerical Simulation of O-Ring Sealing Performance

### 3.1. Irradiation Effects on Material Properties

The sealing properties of rubber materials are highly dependent on their mechanical properties. Irradiation can degrade the material’s properties, which can be evaluated by the mechanical property parameters. No theoretical model exists to describe the quantitative relationship between the irradiation dose and the degradation of rubber materials. Experimental tests are the main approach for obtaining relevant data, and a number of methods for testing the typical mechanical parameters have been reported.

The focus of this paper is the general application of measurement data related to mechanical parameters rather than the data collection. Therefore, typical mechanical property data of EPDM materials obtained from experimental measurements in the literature [14] were used (Table 1). The tested EPDM materials had high irradiation resistance, and their mechanical properties were more influenced by the total irradiation dose than the irradiation dose rate.

Table 1 indicates that the Young’s modulus of the EPDM material increases significantly and the elongation at break decreases significantly with the radiation dose. Thus, irradiation causes the material to become brittle, and the sealing performance of the rubber declines. However, regarding the compression set, the resilience of the material either improves or remains stable as the dose increases. Several studies evaluating sealing properties used the compression set as an indicator of the sealing performance. A low compression set was more conducive to blocking leaks. Therefore, it is difficult to conduct an accurate assessment of the sealing performance based solely on mechanical performance parameters.

**Figure 8:**
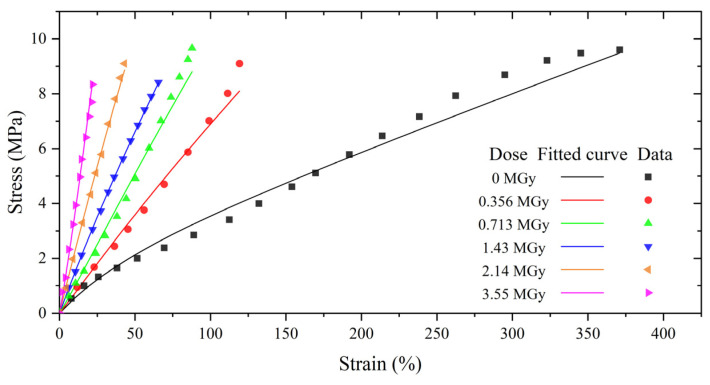
Stress–strain curves of EPDM for different absorbed doses based on the M-R model. (The experimental data were obtained from Ref. [14].)

**Figure 9:**
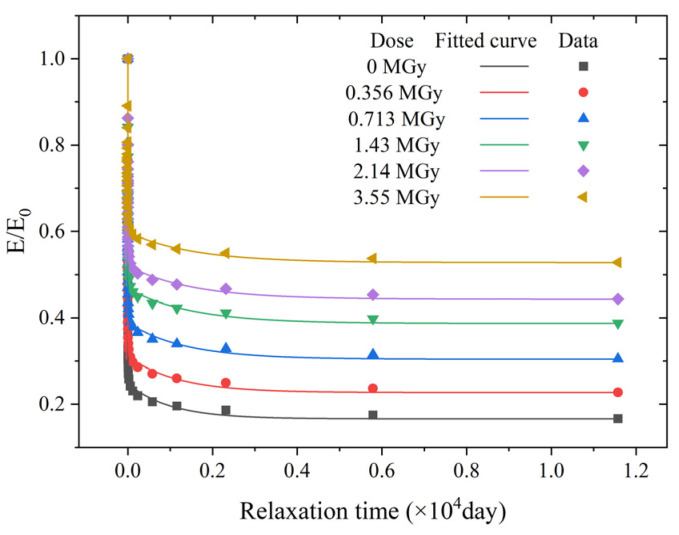
Relaxation curves of EPDM for different absorbed doses based on the five-term Prony series. (The experimental data were obtained from Ref. [14].)

The Mooney–Rivlin (M-R) model was used to describe the hyperelastic properties of the EPDM material, and the generalized Maxwell model in the form of the Prony series was used to describe the viscoelastic properties. The model coefficients were based on experimental measurements, and different dose levels corresponded to different model coefficients. The stress–strain curves and modulus relaxation curves for different absorbed doses of EPDM based on the hyperelastic and viscoelastic models are shown in Figure 8 and Figure 9, respectively. The experimental data were obtained from the literature. The M-R model and the five-term Prony series accurately characterize the hyperelastic and viscoelastic behavior of EPDM at different absorbed doses.

The effect of the absorbed dose on the sealing performance of the EPDM O-rings was investigated numerically based on the constitutive and relaxation models verified in Figure 8 and Figure 9. The validity of the calculated leakage rate was verified by comparing the results with those from the vacuum leak test.

### 3.2. Simulation and Validation of Vacuum Leak Tests

Battini et al. [14] measured the vacuum leakage rate of O-rings exposed to different irradiation doses and different compression rates to determine the O-rings’ sealing performance. The compression rate (*CR*) was defined by the following equation:

(10) $$CR=\frac{d_{s}-d_{s,m}}{d_{s}}\times 100\%$$

where $d_{s}$ is the cross-sectional diameter of the undeformed O-ring, and $d_{s,m}$ is the minimum cross-sectional diameter of the O-ring after compression. A leak test was carried out using a helium leak detector and customized holding devices to achieve different compression rates. Four irradiation doses and six compression rates were considered. The initial leakage rate was 5 × 10^−7^ mbar L/s, and the test objective was to determine the minimum compression rate, CR_lim_, resulting in no leakage of the O-rings at different absorbed doses. The smaller the CR_lim_ value, the better the sealing performance of the O-ring.

**Figure 10:**
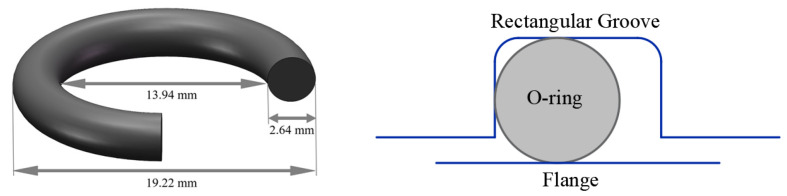
Geometry of the O-ring and the groove used for the simulation.

**Figure 11:**
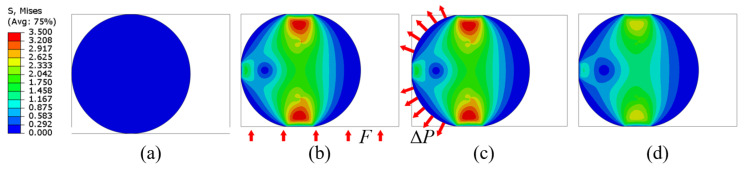
Simulation steps of the vacuum leakage test (at an absorbed dose of 3.55 MGy): (**a**) assembly, (**b**) compression, (**c**) vacuum, and (**d**) relaxation (300 s).

**Figure 12:**
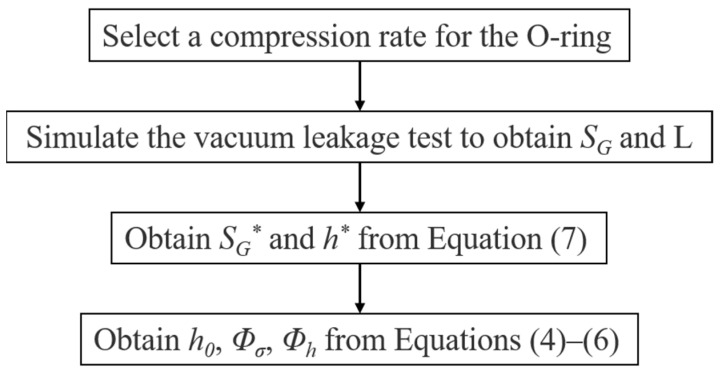
Flowchart for calculating the leakage rate.

In this work, similar leakage tests were carried out using numerical methods. The difference was that the CR_lim_ values were derived from the calculated results of the leakage-rate prediction model. In the numerical experiments, the structural dimensions (see Figure 10), irradiation doses, and test procedures were consistent with those of the vacuum leakage tests performed by Battini et al. [14]. Figure 11 illustrates the numerical simulation steps of the leakage test, including (a) assembly, (b) compression, (c) vacuum, and (d) relaxation (300 s), where *F* is the applied sealing force and Δ*P* is the driving force on the O-ring under differential medium pressure. The contact stress, *S_G_*, and the contact width, L, between the O-ring and the sealing surface were obtained from the simulation results. The leakage-rate prediction model was used to obtain the leakage rate for different compression rates. The flowchart for calculating the leakage rate is shown in Figure 12.

**Figure 13:**
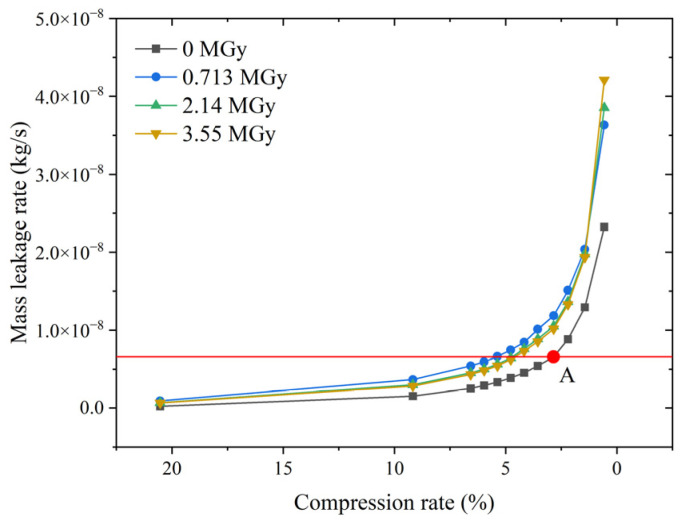
Leakage rates derived from the simulated vacuum leakage test.

**Figure 14:**
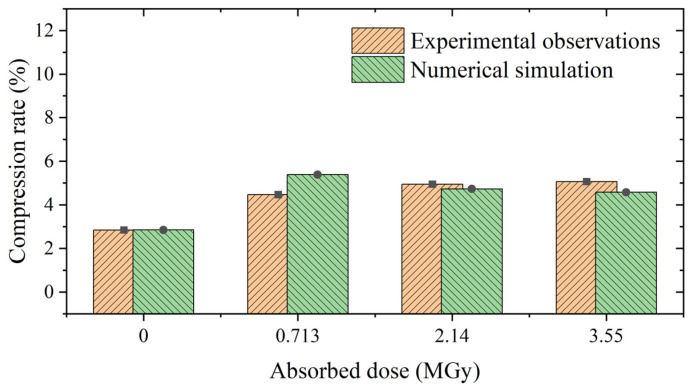
Comparison of calculated and experimental values of CR_lim_.

Figure 13 shows the results of the numerical simulation of the vacuum leakage test. Unlike Battini et al. [14], who expressed the leakage rate in mbar L/s, we express our results in kg/s. When the volume of the container is unknown, the two results cannot be directly compared. However, experiments or simulations can be used to obtain a minimum compression rate, using a leakage rate threshold. We achieved this by using the CR_lim_ of the non-irradiated EPDM O-ring as the calibration point (point A in Figure 13). If the leakage rate at point A is the leakage threshold, the CR_lim_ of the O-ring can be derived at other absorbed doses. A comparison of the measured and simulated CR_lim_ values of the O-ring for different absorbed doses is shown in Figure 14.

The performance of the leakage-rate prediction method is illustrated in Figure 13 and Figure 14. As shown in Figure 13, the leakage rate increases rapidly after reaching the minimum compression rate, CR_lim_, indicating that the leakage rate threshold is appropriate. Figure 14 shows that the CR_lim_ of the non-irradiated O-ring is smaller than that of the irradiated O-ring, and the CR_lim_ differs for different absorbed doses. This result suggests a significant difference in the sealing performance between irradiated and non-irradiated materials, but the absorbed dose does not significantly affect the sealing performance of the irradiated material. These results are in good agreement with the experimental observations reported by Battini et al. [14]

**Figure 15:**
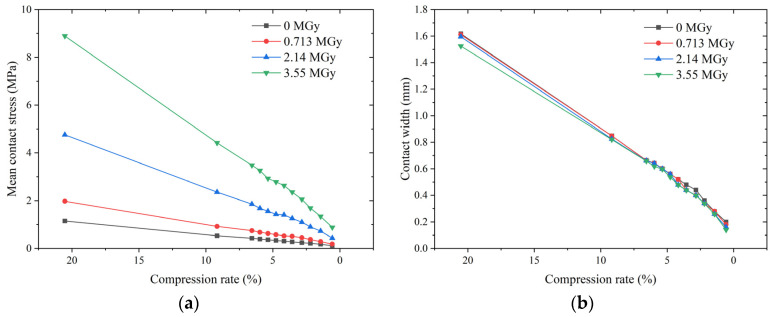
Effect of the compression rate on the mean contact stress and contact width of O-rings for different absorbed doses: (**a**) mean contact stress and (**b**) contact width.

Typically, the compression set is used as one of the indicators to evaluate the sealing performance of a material. A lower compression set means a more stable elastic response (i.e., recovery upon unloading), which usually indicates the better sealing performance of the material. Using the compression set data in Table 1 to evaluate the EPDM, it was expected that the irradiated O-rings would have a superior sealing performance, as their compression set was much lower than that of the non-irradiated O-rings. However, tests and simulations have shown counter-intuitive results, with irradiated EPDM O-rings exhibiting more leakage than the non-irradiated one. Battini et al. [14] explain that the simultaneous increase in material stiffness at high dose levels requires higher compression rates to ensure a no-leak condition. However, this does not explain why, at the same compression rate, the irradiated O-rings have a higher leakage rate than the non-irradiated one. Figure 15 shows the contact state of the O-rings at the same compression rate. As shown in Figure 15a, irradiated O-rings are subjected to greater sealing forces (indicated by the mean contact stress) to achieve the same compression rate. Meanwhile, Figure 15b shows that the contact width is dominated by the compression rate and is almost independent of the dose. These results imply that an increase in material stiffness leads to an enhanced sealing contact state for the same compression rate. Obviously, the degradation of leakage behavior due to irradiation effects cannot be adequately explained through a mechanical analysis alone.

**Figure 16:**
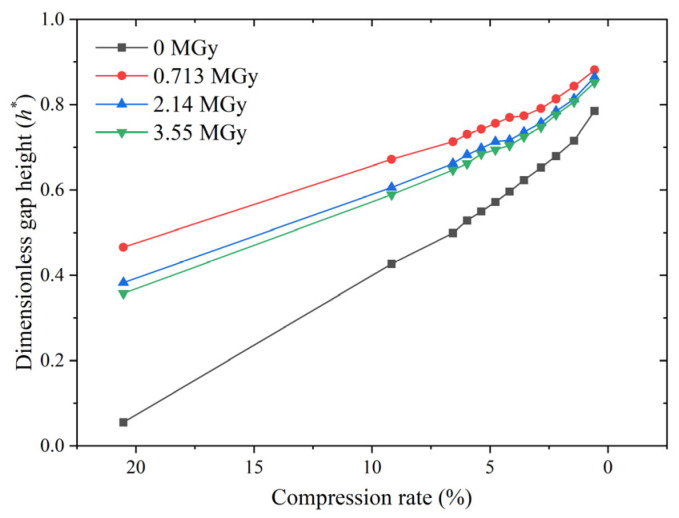
The effect of the compression rate on the interfacial gap height of O-rings for different absorbed doses.

The interfacial leakage model provides a good explanation for the leakage performance. Equation (4) indicates that the interfacial leakage rate primarily depends on the dimensionless gap height when the roughness characteristics are known. The effect of irradiation on the interfacial gap height depends on two conflicting aspects. First, an increase in material stiffness increases the contact stress at the same compression rate, thus reducing the gap height. Second, a large increase in the Young’s modulus due to material embrittlement causes more difficulty in compressing the asperities, thus preventing a reduction in the gap height. The dimensionless gap height depends on the combined effect of these two factors. Figure 16 shows the effect of the compression rate on the gap height for different absorbed doses. The trend of the gap height closely matches that of the leakage rate: a difference exists between the gap heights of the irradiated and non-irradiated O-rings, and there is a negligible difference the gap heights of O-rings for different irradiation doses.

Figure 16 also shows that the higher the compression rate, the greater the difference in gap height between the non-irradiated and irradiated O-rings. However, since the leakage rates of all O-rings are very low at high compression rates, the differences are non-significant. In this case, the risk of seal failure due to a high absorbed dose is related to structural cracking caused by material embrittlement, which can be predicted by numerical calculations. However, this aspect is not discussed in this paper.

## 4. Irradiation Effect on the Sealing Performance of O-Rings at High Pressure

Investigating the effect of irradiation on the high-pressure sealing performance of O-rings is particularly critical when considering the pressure boundary integrity of the NPP containment in an accident. The proposed leak rate prediction method is applied to investigate the high-pressure sealing behavior of irradiated and non-irradiated O-rings. Unless otherwise stated, the primary calculation parameters for the O-rings are listed in Table 2.

### 4.1. Analysis of Mechanical Behavior

**Figure 17:**
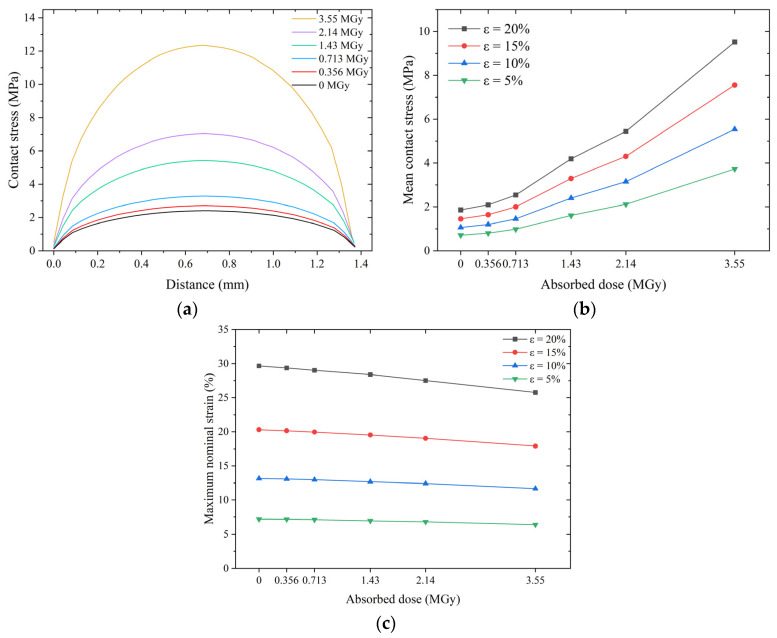
Effect of irradiation on pre-compressed O-rings: (**a**) contact stress distribution (ε = 20%), (**b**) mean contact stress, and (**c**) maximum nominal strain.

A high pre-compression rate (e.g., 20%) is usually required to ensure a high sealing performance of O-rings under high-pressure conditions. Figure 17a shows the contact stress distribution of the O-ring for different absorbed doses at a compression rate of 20%. The higher the absorbed dose, the higher the sealing force required for the O-ring to achieve the same compression rate due to a significant increase in stiffness. The maximum contact stress of the O-ring at an absorbed dose of 3.55 MGy is 12 MPa, whereas that of the non-irradiated O-ring is only 2 MPa.

Figure 17b shows that the trend of mean contact stress with the absorbed dose is very similar for O-rings with different compression rates. At low doses (less than 0.713 MGy), the mean contact stress increases slowly with the absorbed dose; at higher dose levels (greater than 0.713 MGy), the mean contact stress increases linearly with the absorbed dose.

Figure 17c shows the effect of the irradiation dose on the maximum nominal strain of the O-ring. At the same compression rate, the maximum strain decreases only slightly with an increase in the absorbed dose, and the maximum rate of decrease (at a 20% compression rate) does not exceed 4%. However, it should be noted that according to the data in Table 1, the strain at break of EPDM at a dose of 3.55 MGy is only 23 ± 3%, which is much lower than the 370% at 0 MGy. This result indicates that materials exposed to high absorbed doses are prone to brittle cracking at high compression rates.

**Figure 18:**
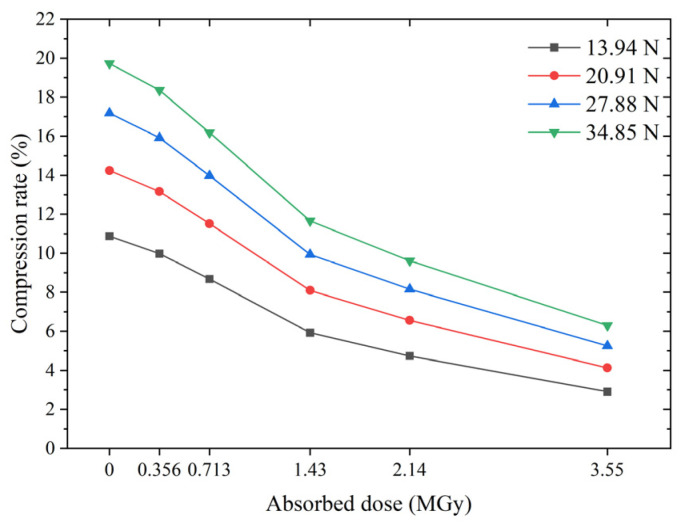
Compression rate versus absorbed dose for a given sealing force of the O-ring.

It should be noted that there is another pre-compression mode for O-rings; that is, the O-ring is compressed at a fixed sealing force. In such a situation, the compression rate of the irradiated O-ring decreases significantly with the increasing dose due to the higher stiffness, as shown in Figure 18. The higher the sealing force, the more pronounced the decrease in the compression rate. At a sealing force of 34.85 N, the compression rate of the non-irradiated O-ring is 20%, whereas that of the O-ring with a dose of 3.55 MGy is only about 6%. In this case, the risk of brittle cracking due to a high dose is low. However, the risk of seal failure has significantly increased.

### 4.2. Analysis of Leakage Behavior

**Figure 19:**
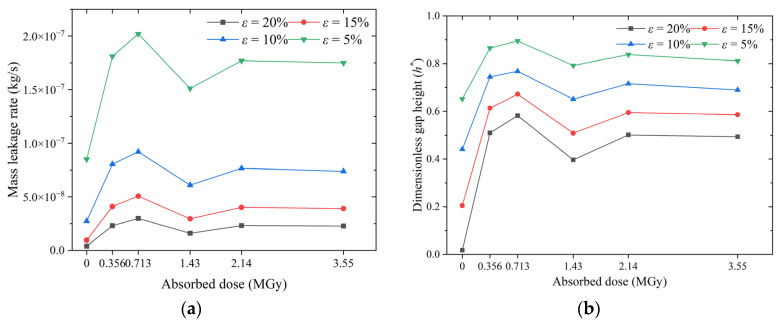
Effect of absorbed dose on O-ring leakage behavior at different compression rates: (**a**) mass leakage rate and (**b**) dimensionless gap height.

The effect of the absorbed dose on the leakage behavior of O-rings at different compression rates is shown in Figure 19. At the same absorbed dose, an increase in the compression rate causes an increase in the contact stress and a subsequent decrease in the leakage rate. When the compression rate is increased from 5% to 10%, the leakage rate of the O-ring decreases by a factor of about two; when it is increased to 20%, the leakage rate decreases by a factor of about five. However, the relationship between the contact stress and the leakage rate of O-rings with different absorbed doses does not conform to this rule. At the same compression rate, the contact stress of the O-rings increases monotonically with the dose (see Figure 17b); however, the leakage rate increases non-monotonically. As shown in Figure 19a, the leakage rate of the non-irradiated O-rings is always the lowest at all compression rates, whereas that of the irradiated O-rings increases, decreases, increases, and then gradually stabilizes as the dosage increases. Altering the compression rate changes the leakage rate of all O-rings, but the trend with the dose remains the same: the leakage rate is the highest at a dose of 0.713 MGy and lowest at a dose of 1.43 MGy (except for the non-irradiated O-rings).

The non-monotonic increase in the leakage rate with the dose can be explained by the response of the dimensionless gap height (Figure 19b). The gap height depends on the indentation depth on the sealing surface. An increase in material stiffness due to irradiation has both positive and negative effects on the indentation depth. The increase in the interfacial contact stress is a positive effect, whereas the increased difficulty of micro-contact deformation is a negative effect. At lower absorbed doses, i.e., 0.356 MGy and 0.713 MGy, the negative effect is greater than the positive effect: the gap height increases with the increasing dose, and the leakage rate rises. At a dose of 1.43 MGy, the positive effect predominates; the gap height and the leakage rate decrease with the increasing dose. As the dose is further increased, i.e., 2.14 and 3.55 MGy, the positive and negative effects cancel each other out, and the leakage rate stabilizes. Figure 19a,b show that the compression rate prevents the O-ring’s deterioration due to the irradiation effect.

**Figure 20:**
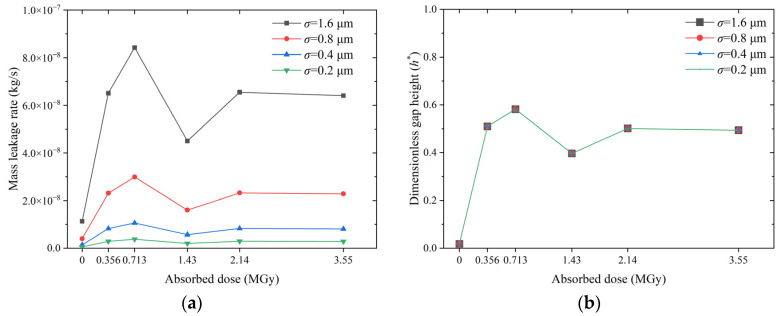
Effect of absorbed dose on O-ring leakage behavior at different roughness levels: (**a**) mass leakage rate and (**b**) dimensionless gap height (The four curves are overlaps).

Figure 20 presents the effect of the absorbed dose on the leakage rate and dimensionless gap height of the O-ring for different roughnesses. The results in Figure 20a show that an increase in roughness causes a significant increase in the leakage rate. The leakage rate at *σ* = 1.6 μm is almost three times higher than at *σ* = 0.8 μm and an order of magnitude higher than at *σ* = 0.2 μm. Figure 20b shows that the roughness has a negligible effect on the dimensionless gap height and, therefore, only a slight effect on the leakage rate as the dose increases. However, a larger roughness results in a larger initial gap height, *h*_0_, and a larger $Q_{P}$ in Equation (1). Thus, an increase in roughness amplifies the difference in sealability caused by the radiation effect, whereas a decrease in roughness suppresses the effect of the radiation influence on the sealing performance.

**Figure 21:**
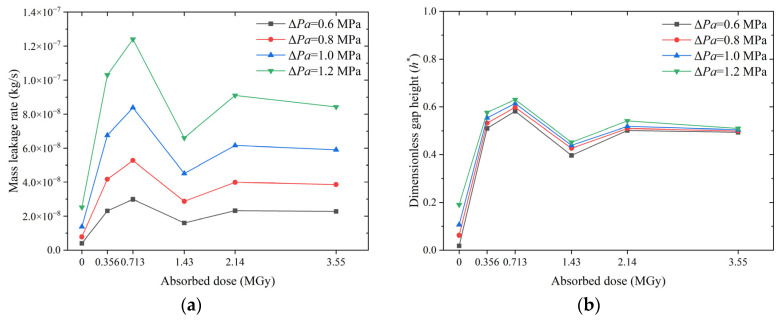
Effect of absorbed dose on O-ring leakage behavior at different pressures of the medium: (**a**) mass leakage rate and (**b**) dimensionless gap height.

Figure 21 shows the effect of the medium’s pressure on the sealing performance of irradiated and non-irradiated O-rings. The pressure affects the leakage rate; the higher the pressure, the higher the leakage rate, as shown in Figure 21a. The leaking medium enters the contact interface and offsets some of the contact stress, resulting in an increase in the dimensionless gap height, as shown in Figure 21b. Since the contact pressure at the interface of the non-irradiated O-ring is minimal (only about 2 MPa; Figure 17b), which is comparable to the pressure considered (0.6 MPa to 1.2 MPa), the effect of the pressure on the dimensionless gap height is more pronounced. The O-ring with an absorbed dose of 3.55 MGy has an interfacial contact stress of approximately 10 MPa (see Figure 17b), which is much higher than the medium’s pressure considered (0.6 MPa to 1.2 MPa), and its dimensionless gap height is very little affected by the medium’s pressure. Therefore, the shape of the leakage rate versus absorbed dose curves is independent of the pressure of the medium.

These results demonstrate that the leakage behavior of the O-ring depends not only on the material properties but also on the compression rate, roughness, medium pressure, and other parameters. The leakage rate is the most suitable evaluation indicator of sealing performance because it considers the other factors.

### 4.3. Assessment of the Sealing Performance

The proposed leakage rate prediction method is applied to evaluate the O-ring’s sealing performance: the maximum allowable leakage rate is used as a criterion to determine the maximum pressure level that the O-ring can be subjected to at different absorbed doses.

The ISO 5208 standard [36] classifies industrial valves into 10 classes with different maximum allowable leakage rates. Here, the leakage criterion for Class C valves is selected, and the volumetric leakage rate is converted to a mass leakage rate to obtain the maximum allowable leakage rate (*Q_lim_*) of 4.81 × 10^−8^ kg/s for a 13.94 mm diameter O-ring under consideration.

**Figure 22:**
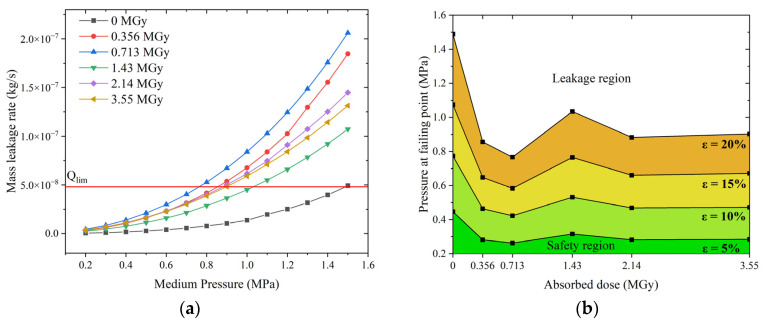

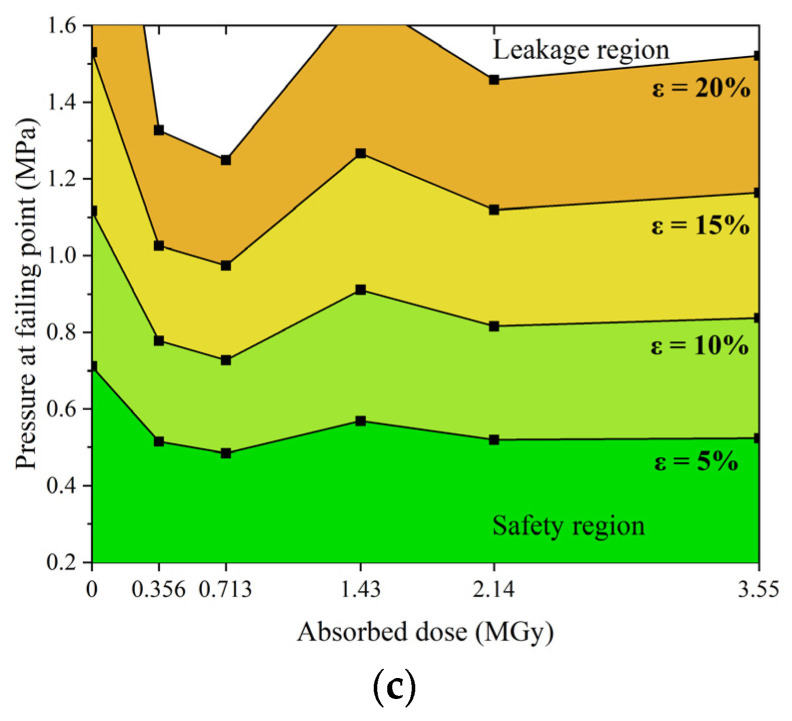
Influence of the radiation dose on O-ring sealing performance under high pressure: (**a**) leakage rate versus pressure, (**b**) classification of sealing performance for *σ* = 0.8μm, and (**c**) classification of sealing performance for *σ* = 0.4μm.

Figure 22 shows the results of the sealing performance of the O-rings at high pressures. The proposed prediction method was used to analyze the leakage behavior of the O-rings at different doses. The resulting leakage rate at medium pressure is shown in Figure 22a. Next, *Q* ≤ *Q_lim_* was used as the sealing evaluation criterion to derive the failure pressure, which was defined as the pressure at which the leakage rate of the O-ring exceeded the allowable value. The failure pressure values for O-rings at different absorbed doses for different compression rates are listed in Table 3. The results were used to create the classification of the sealing performance in Figure 22b.

As shown in Figure 22b, the results were divided into upper and lower zones based on the failure pressure. The upper region is the leakage zone, and the lower region is the safety region (sufficient sealing). The failure pressure curves differ for different compression rates. For example, the failure pressure curve for *ε* = 5% is much lower than for *ε* = 20%. If the desired failure pressure is known, Figure 22b can be used to determine the minimum compression rate of the O-ring. For a containment with a design pressure of 0.5 MPa, the minimum compression of the O-ring should exceed 10%. In severe accidents where the pressure in the containment may peak at 0.8 MPa, the compression of the O-ring should exceed 20%. Figure 22 shows the results for a composite roughness of 0.8 μm on the sealing surfaces. The failure pressure curve is shifted upwards when the roughness is 0.4 μm (Figure 22c).

## 5. Conclusions

1. A leakage rate prediction model for O-rings was developed. The model was derived using mesoscopic interfacial gap flow simulations. The gap height was used as a critical parameter to couple the gap flow analysis with the mechanical simulation to predict the leakage rate of the irradiated O-ring under operating conditions. The model was proved to be an accurate predictor of the onset of leakage and was validated by a vacuum test.
2. The proposed model was applied to predict the leakage behavior of O-rings after irradiation. Although the mechanical properties of the material degraded monotonically with an increase in the absorbed dose, the leakage rate increased non-monotonically. The model-based analysis shows that this non-monotonicity can be accurately indicated by the dimensionless gap height, thus revealing that it is the result of the combined effect of contact interface micro-deformation and elastomer macro-deformation.
3. When all other factors remained the same, the sealing performance of the O-rings was always the worst at a dose of 0.713 MGy and optimum/best at a dose of 1.43 MGy at all irradiation doses. The high compression rate maintained the seal, even at an irradiation dose of 3.55 MGy, but the O-ring may become brittle. In contrast, the non-irradiated O-ring exhibited a much better sealing performance than the irradiated O-ring.
4. A method was developed to classify the sealing performance by using the maximum allowable leakage rate as an indicator. It can be used to determine the appropriate compression rate of an O-ring based on the design requirements of the site (pressure and exposed dose). It can also be used to assess the safety of a seal based on the pressure level under accident conditions.
5. The models and methods presented in this paper can be used not only to assess the safety limits of irradiated materials but also their service life. The only difference is that Table 1 needs to be supplemented with data reflecting the effects of ageing. Both the life analysis and safety assessment are topics of great interest for elastomers used in nuclear power plants, and further work can be carried out on the basis of this work.

## Figures and Tables

**Table 1:** Mechanical properties and absorbed dose of a specific EPDM [14].

Absorbed Dose (MGy)	Young’s Modulus (MPa)	Strain at Break (%)	Tensile Strength (MPa)	Compression Set (%)
3.55	37.07	23 ± 3	8.3 ± 1	/
2.14	21.11	43 ± 6	9.1 ± 0.7	5.4 ± 0.3
1.43	12.68	65 ± 6	8.4 ± 0.6	4.8 ± 0.3
0.713	10.98	88 ± 7	9.7 ± 1	5.2 ± 0.3
0.356	7.30	120 ± 7	9.1 ± 0.6	3.9 ± 0.3
0	2.63	370 ± 40	9.6 ± 0.9	9.0 ± 0.3

**Table 2:** Primary parameters for the leakage prediction of O-rings.

Parameters	Values
Cross-sectional diameter (mm)	2.64
Internal diameter (mm)	13.94
Irradiation dose (MGy)	0, 0.356, 0.713, 1.43, 2.14, 3.55
Surface roughness (μm)	0.8
Compression ratio (%)	20
Pressure difference (MPa)	0.6

**Table 3:** Failure pressure of O-rings for different absorbed doses (MPa).

	Absorbed Doses (MGy)	0	0.356	0.713	1.43	2.14	3.55
Compression Rate							
ε = 5%		0.45	0.28	0.26	0.32	0.28	0.28
ε = 10%		0.77	0.46	0.42	0.53	0.47	0.47
ε = 15%		1.07	0.64	0.58	0.76	0.66	0.67
ε = 20%		1.49	0.86	0.77	1.03	0.88	0.90

## Data Availability

Not applicable.
